# Evaluation of a strict protocol approach in managing women with severe disease due to hypertension in pregnancy: A before and after study

**DOI:** 10.1186/1742-4755-2-7

**Published:** 2005-09-30

**Authors:** Hennie Lombaard, Robert C Pattinson, Fèbè Backer, Peter Macdonald

**Affiliations:** 1 Department of Obstetrics and Gynaecology, Kalafong Hospital, Private Bag X396, Pretoria 0001, South Africa; 2 MRC Maternal and Infant Health Care Strategies Research Unit and Obstetrics and Gynaecology Department, University of Pretoria, South Africa

## Abstract

**Background:**

To evaluate whether the introduction of a strict protocol based on the systemic evaluation of critically ill pregnant women with complications of hypertension affected the outcome of those women.

**Method:**

Study group: Indigent South African women managed in the tertiary hospitals of the Pretoria Academic Complex. Since 1997 a standard definition of women with severe acute maternal morbidity (SAMM), also referred to as a Nearmiss, has been used in the Pretoria Academic Complex. All cases of SAMM and maternal deaths (MD) were entered on the Maternal Morbidity and Mortality Audit System programme (MaMMAS). A comparison of outcome of severely ill women who had complications of hypertension in pregnancy was performed between 1997–1998 (original protocol) and 2002–2003 (strict protocol). Data include women referred from outside the Pretoria Academic Complex area to the tertiary hospitals.

**Results:**

Between 1997–1998 there were 79 women with SAMM and 18 maternal deaths due to complications of hypertension, compared with 91 women with SAMM and 13 maternal deaths in 2002–2003. The mortality index (MI) declined from 18.6% to 12.5% (OR 0.62, 95% CI 0.27–1.45). Statistically significant fewer women had renal failure (RR 0.37, 95% CI 0.21 – 0.66) and cerebral complications (RR 0.52, 95%CI 0.34 – 0.81) during the second period, and liver dysfunction (RR 0.27 95%CI 0.06 – 1.25) tended to be lower. However, there tended to be an increase in the number of women, who had immune system failure (RR 4.2 95%CI 0.93 – 18.94) and respiratory failure (RR 1.42 95%CI 0.88 – 2.29) although it did not reach significance. Cardiac failure remained constant (RR 0.84 95%CI 0.54 – 1.30).

**Conclusion:**

The strict protocol approach based on the systemic evaluation of severely ill pregnant women with complications of hypertension and an intensive, regular feedback mechanism has been associated with a reduction in the number of patients with renal failure and cerebral compromise.

## Background

Complications due to hypertensive disease in pregnancy are the most common direct cause of maternal death (MD) in South Africa [1,2]. They are also the most common cause of acute severe morbidity in a survey of three clearly defined geographical areas in South Africa [3]. The final and contributory causes of deaths were most commonly cerebral complications (40% and 50%), cardiac failure (40% and 35%), respiratory failure (15% and 16%) and renal failure (10% and 18%) in 1998 and 1999–2001 respectively [1,2]. Any strategy for improving the outcome of severely ill pregnant women with complications due to hypertension would have to address ways of preventing these organ systems from failing.

In 1997, the Pretoria Academic Complex initiated the use of a standard definition of severe acute maternal morbidity (SAMM) [4] and the routine collection of data since. Table 1 shows the definition for organ system dysfunction for each specific organ system. After the initial survey in 1997–1998, protocols were reviewed and a new approach to managing severely ill pregnant women was introduced. This system was based on the systematic routine evaluation of all organ systems and, where an abnormality was detected, this served as a trigger to further investigate and support that organ system [5]. This policy was adopted for managing critically ill pregnant women with specific common conditions and as a group became known as "the strict protocols".

**Table 1:** The criteria for a near-miss case for each specific organ system according to Mantel.

Organ system-based	Markers
Cardiac dysfunction	Pulmonary oedema Cardiac arrest
Vascular dysfunction	Hypovolaemia requiring ≥ 5 units of blood products
Immunological dysfunction	ICU admission for sepsis Emergency hysterectomy for sepsis
Respiratory dysfunction	Intubation and ventilation for any reason other than general anaesthesia Oxygen saturation of less than 90% for more than 60 min The ratio of the partial pressure of oxygen in arterial blood to the percentage oxygen in inspired air is ≤ 3 (paO2/FiO2 ≤ 3)
Renal dysfunction	Oliguria, ≤ 400 ml/24 hr that does not respond to careful fluid replacement or attempts at inducing with dopamine or infusion Acute deterioration in urea > 15 mmol/l or of creatinine to > 400 mmol/l
Liver dysfunction	Jaundice in the presence of pre-eclampsia
Metabolic dysfunction	Diabetic keto-acidosis
Coagulation dysfunction	Acute thrombocytopenia requiring a platelet transfusion
Cerebral dysfunction	Coma lasting > 12 hours Subarachnoid or intracerebral haemorrhage

The implementation of new policies can be difficult to achieve [6]. The change in policy was effected by regular reinforcement at daily audit meetings at the individual hospitals and weekly departmental meetings where all cases of SAMM and MD were discussed. New trainees were introduced to the protocols from the start, face-to-face meetings were held when problems were detected, and special training sessions were introduced to stress specific points. Finally, a report was compiled and presented to the provincial administration with specific recommendations appropriate to the region. There has been ongoing regular contact since then.

This study examines the effect of the introduction of this strict protocol on the outcome of critically ill pregnant women with complications of hypertension in pregnancy and attempts to identify where further research or change in policy is required.

## Methods

The Pretoria Academic Complex consists of two academic hospitals (Pretoria Academic and Kalafong) and two district hospitals (Mamelodi Day and Pretoria West). The area receives referrals from other areas within Gauteng Province and the surrounding provinces. The Pretoria Academic Complex serves a population of 3 million people. The population served is mainly an indigent general South African population. The female population was 554 000 in 1996 with an annual growth of 10%, the fertility rate for Gauteng is 2.3% for women aged 15 to 49 and the current pregnancy rate is 2.2% [17]. The number of births within the immediate area has increased by twenty percent over the last five years and is currently approximately 15 000 per year. For the period of 1997/1998, the total number of births was 27 025, the maternal mortality ratio was 133.2/100 000 births and the perinatal mortality rate was 37/1000 for babies weighing more than 1000 gr. For the period of 2002/2003 the total number of births was 32 814, the maternal mortality ratio was 115.8/100 000 births and the perinatal mortality rate for babies weighing more than 1000 gr was 27/1000 births.

The criteria for SAMM have been defined by Mantel *et al*. [4] as a woman with organ system dysfunction or failure who would probably have died if un- or inadequately treated. The criteria are based on clinical evaluation with limited specific investigations that are readily available at all regional, secondary and higher level hospitals.

Data on women with SAMM and MD were collected every morning at the respective hospitals and a near miss form was completed for each woman with SAMM and the maternal death notification form for all maternal deaths. The data were entered into the Maternal Morbidity and Mortality Audit System (MaMMAS) database, developed by Johan Coetzee (Simply Software). Data from 1997/1998 (initial project) [3] served as the platform for developing the database. This initial project compared Maternal Mortality and Nearmiss to determine if disease pattern was the same. In the second time period (2002/2003) the strict protocol had been implemented and became established.

Standard statistical techniques were used to compare the two time periods. The chi square test was used to compare categorical data. The outcome measures were the Mortality Index, defined as the number of maternal deaths divided by the sum of women with SAMM and maternal deaths, expressed as a percentage [7]. The criteria for each organ system dysfunction/failure were specified in the definition of a woman with SAMM [4] and the rates of each organ system dysfunction were compared between the two time periods. The relative risk was used to compare the two time periods as it gives a more realistic indication of the changes.

During the first time period (1997/1998) there was no strict protocol available and standard care was provided. This consisted of magnesium sulphate for eclamptic fits and hydralazine 1,25 mg ivi every 15 min for the acute treatment of hypertension if the diastolic BP was ≥ 110 mmHg. A fluid balance chart was not routinely kept and the management of the patient was mainly based on the opinion of the attending consultant.

In contrast, the strict management protocol, implemented during the second time period (2002/2003) included the following:

### Stabilization

The stabilization of the patient is summarised in Table 2.

**Table 2:** Summary of stabilising the severely ill women with complications of Hypertension after admission to a High Care Obstetrics unit

Organ system	Acute management	Maintenance	Management of complications
Fluid management	Start IV line give 300 ml fluid bolus: 100 ml Ringers lactate 200 ml normal saline with loading dose of magnesium sulphate Urinary Catheter	Give Ringers lactate 125 ml/hr iv. Start a fluid balance chart	If poor output repeat fluid bolus. If still poor output and positive fluid balance start low-dose dopamine infusion
Magnesium Sulphate	4 g magnesium sulphate in 200 ml saline over 20 min iv 5 g magnesium sulphate with 1 ml lignocaine im in each buttock	Maintenance: 5 g four hourly iv Check before next dosage: Urine output > 30 ml/hr Tendon reflexes present Respiratory rate more than 16/min	In case of magnesium sulphate overdose give calcium gluconate
Blood pressure control	Repeat blood pressure after 20 min and if diastolic ≥ 110 or systolic ≥ 160 treat according to the antihypertensive drug protocol	Use either nifedipine or labetolol	
Neurological status	If still confused check saturation and blood pressure	Abnormal saturation: Give oxygen via mask Abnormal blood pressure: treat with appropriate drugs	If both are normal: give haloperidol

### Systemic Evaluation

Table 3 summarizes the systemic evaluation of the mother.

**Table 3:** Summary of the systemic evaluation and special investigations of critical ill women with complications of hypertension

Organ system evaluated	Clinical examination	Special investigations
Central nervous system	Glasgow coma scale Lateralising signs Reflexes Pupil reflexes	If any abnormalities consider CT Scan
Respiratory system	Respiratory rate Blood gas Check for dullness on percussion, crepitations or wheezes	If any abnormalities do blood gas and Chest X-ray
Cardiovascular system:	Pulse, Blood pressure Heart sounds Heart size Radio-femoral delay	
Gastro intestinal system:	Check for epigastric tenderness, hepatomegaly	Check AST and for jaundice. 4 hourly blood glucose test if raised AST
Renal system:	Check for renal angle tenderness, macroscopic hematuria Listen for murmurs over the renal artery	Check creatinine and fluid balance. If signs of kidney dysfunction do full kidney function tests
Haematological system:	Check for anaemia, purpera, bleeding tendency	Check hematocrit and platelets
Immune system:	Body temperature Check for generalized lymphadenopathy, splenomegaly, signs of immune system failure	Voluntary counselling and HIV testing if CD4 and ESR above 100
Musculosceletal System	Check for signs of DVT Check for spinal problems that might influence the type of anaesthesia	
Gynaecological system:	Abdominally: measure symphysis-fundus height, lie & position of the foetus, check for uterine tenderness or contractions, estimate foetal weight, measure amniotic fluid, check for foetal heart rate Vaginal exam: assess the Bishop score	
Fundoscopy:	Check for silver wiring, papillar oedema and signs of bleeding	

### Foetal Evaluation

An ultrasonographic evaluation of the foetus was performed, once the mother had been stabilised, and included the following:

• Estimated foetal weight

• Doppler of the umbilical artery

• Amniotic fluid index

• Transcerebellar diameter

• Middle cerebral artery Doppler

• Ductus venosus waveform

• Detection of possible structural abnormalities

If the expected foetal weight was more than 800 g or the foetus was known to have a gestational age of 28 weeks or more, the foetus was regarded as viable. In these cases corticosteroids were administered and the foetal heart rate pattern was monitored six hourly with a cardiotocograph (CTG).

After all this information had been gathered a management plan was formulated. In most cases the laboratory blood results (initially only aspartate-amino transferase (AST), creatinine, haematocrit and platelets) were available within the hour and during the stabilisation phase of the patient. To make a final decision four questions needed to be answered by the clinician, namely:

1. Is it safe for the mother to continue the pregnancy?

2. Is it safe for the foetus to continue the pregnancy?

3. What is the risk to neonate if the foetus was born?

4. What is the risk to the mother if the foetus was born?

In case of expectant management, the woman was transferred to a high care (high dependency unit) obstetric unit, with daily evaluation by the registrar and routine blood testing (haematocrit, platelets, creatinine and AST) twice weekly. Cardiotocogram (CTG) (6 hourly) and ultrasound (two-weekly) were used to assess foetal wellbeing and growth. If there was concern about foetal growth, ultrasound was repeated more often [10].

The indications for delivery were:

• Foetal distress

• Intra uterine death

• Expected birth weight more than 2 kg

• Expected birth weight less than 500 g

• Maternal organ system failure

• Uncontrollable hypertension

• Eclampsia

• Proven foetal lung maturity

• Foetal abnormality

The indications for elective caesarean section were:

• Unfavourable Bishop score (4 or less)

• Foetal distress as diagnosed on the basis of spontaneous decelerations on CTG

• Absent end diastolic flow of the uterine artery on Doppler

• An abnormal ductus venosus waveform. The ductus venosus wave form was regarded as abnormal if it was absent or reversed.

### Antihypertensive drug protocol

Alpha methyldopa was used as the first line oral antihypertensive agent [10] (an initial dose of 500 mg 8 hourly was used increasing to a maximum of 750 mg 8 hourly). In cases of severe hypertension (blood pressure more than 160 mmHg systolic or 110 mmHg diastolic) 10 mg nifedipine [11] was administered orally. Nifedipine (10 mg) has replaced dihydralazine in the strict protocol as it is more effective in controlling blood pressure and is safer for the foetus [11,12]. If the blood pressure remained higher than 160/110 mmHg after one hour a repeat dose of nifedipine was given. If the woman was unable to swallow or had a tachycardia of more than 120 beats per minute, labetolol [13] was administered: the patient was started with 20 mg iv and if the blood pressure remained above 160/110 mmHg after 10 min she would receive 40 mg iv. If her blood pressure still remained above 160/110 mmHg she would receive 80 mg iv. This would be repeated another two times 10 minutes apart if needed.

### Fluid management

After admission to the High Care Obstetric Unit, the woman was maintained on 125 ml/hr Ringers lactate intravenously. A strict input and output chart was recorded by the nursing staff. The attending registrar regularly determined the fluid balance taking into account insensible and blood loss. If the patient was excreting less than 30 ml per hour and was in a negative balance a 300 ml Ringers lactate bolus was administered. If the patient had positive fluid balance a low dose dopamine infusion was started [9].

### Ethical considerations

The Ethics Committee of the Faculty of Health Sciences gave approval for the initial study and the programme remains registered with the Ethics Committee. The hospital administration at each hospital continues to give approval for the audit. Patient information is anonymised once entered into the database.

## Results

In 1997/1998 there were 27,025 births in the Pretoria Academic Complex and 32,814 births in 2002/2003, an increase of 21.4%. This excludes all referrals and patients born in private institutions. The Maternal Mortality Ratio (MMR) for indigent patients from the Pretoria area, dying from complications of hypertension in pregnancy remained unchanged with 9 deaths in 1997/1998 (MMR 33.3/100000 births) and 8 deaths in 2002/2003 (MMR 24.4/100000 births). However the prevalence of women severely ill from complications of hypertension in pregnancy in our population rose from 40 patients (0.15%) in 1997/1998 to 64 patients (0.20%) in 2002/2003. The denominators for referred patients are unknown.

Auditing all patients with complicated hypertension demonstrated that in 1997/1998 there were 79 SAMM and a further 18 maternal deaths compared to 91 women with SAMM and 13 maternal deaths in 2002/2003. The distribution of age and parity in both groups was similar: age less than 20 years (11% and 9.6%), 20 to 29 years (50.6% and 52.8%), 30 to 39 years (28.9% and 31.7%) and primigravid (42.3% and 43.2%).

Table 4 compares the distribution of the different categories of women with severe complications of hypertension in pregnancy. Statistically significantly more women had severe complications following eclampsia during 1997/1998 compared to 2002/2003, but the opposite was true for women with severe complications following HELLP syndrome. It is important to note that the diagnosis of eclampsia or HELLP syndrome does not automatically classify a woman as having severe morbidity as there also has to be organ system dysfunction as defined above. Overall, the Mortality Index declined from 18.6% in 1997/1998 to 12.5% in 2002/2003 (Odds Ratio 0.62, 95% CI 0.27–1.45). There were no differences in Mortality Indices within the individual disease categories.

**Table 4:** Comparison between the sub-categories of complications of hypertension in pregnancy and their Mortality Indices.

	**1997–1998**					**2002–2003**					**p MI**
	**MD**	**SAMM**	**Total**	**%**	**MI**	**MD**	**SAMM**	**Total**	**%**	**MI**	
Chronic Hypertension	1	2	3	3.1	33.3	1	4	5	4.8	20.0	
Proteinuric Hypertension	6	22	28	28.9	21.4	4	30	34	32.7	11.8	0.49
Eclampsia*	9	38	47	48.5	19.1	6	22	28	26.9	21.4	0.52
HELLP**	2	17	19	19.6	10.5	1	35	36	34.6	2.8	0.27
Liver rupture	0	0	0	0.0	0.0	1	0	1	1.0	100.0	

**All Hypertension**	**18**	**79**	**97**	**100**	**18.5**	**13**	**91**	**104**	**100**	**12.5**	**0.32**

MD – Maternal Death; SAMM – Severe acute maternal morbidity; MI – Mortality Index

* – Significant decline in proportion of eclampsia from 1997/8 to 2002/3, p = 0.0026

** – Significant increase in proportion of women with HELLP syndrome 1997/8 to 2002/3, p = 0.026

Table 5 compares the organ system dysfunction/failure for severely ill pregnant women with complications of hypertension. Significantly fewer women had renal failure (34.0% vs 12.5%, RR 0.37, 95% CI 0.21 – 0.66) and cerebral complications (35.1% vs 14.4%, RR 0.52 95%CI 0.34 – 0.81) than before, and liver dysfunction (7.2% vs 1.9%, RR 0.27 95%CI 0.06 – 1.25) tended to be lower. However, there was a trend towards an increase in number of women who had immune system failure (2.0% to 8.7%, RR 4.2 95%CI 0.93 – 18.94) and respiratory failure (21.6% to 30.8%, RR 1.42 95%CI 0.88 – 2.29) in the second period compared to the first although it did not reach significance. Cardiac failure remained constant (30.9% and 26.0% RR 0.84 95%CI 0.54 – 1.30).

**Table 5:** Comparison of the prevalence of organ system dysfunction/failure per severely ill pregnant women with complications due to hypertension.

**Organ system**	**1997–1998**				**2002–2003**				**RR (95% CI)**
	**SAMM **n = 79	**MD **n = 18	**SAMM+MD **N = 97	**% OSD**	**SAMM **N = 91	**MD **N = 13	**SAMM+MD **N = 104	**% OSD**	
Hypovolaemic shock	7	1	8	8.2	5	1	6	5.8	0.7 (0.25 – 1.94)
Respiratory failure	17	4	21	21.6	29	3	32	30.8	1.42 (0.88 – 2.29)
Cardiac failure	25	5	30	30.9	23	4	27	26.0	0.84 (0.54 – 1.30)
Renal failure	29	4	33	34.2	11	2	13	12.5	0.37 (0.21 – 0.66)
Liver failure	5	2	7	7.2	1	1	2	1.9	0.27 (0.06 – 1.25)
Cerebral complications	24	10	34	35.1	9	6	15	14.4	0.52 (0.34 – 0.81)
Haematological dysfunction	25	4	29	29.9	26	1	27	26.0	0.87 (0.56 – 1.36)
Immune system failure*	1	1	2	2.1	6	3	9	8.7	4.2 (0.93 – 18.94)

% OSD – Percentage of severely ill women who developed that organ system dysfunction/failure

Note: A patient can have more than one organ system dysfunction/failure

* Fisher exact: 2 sided 0.060

: 1 sided 0.038

The average number of organ systems that failed or were dysfunctional for all critically ill women were 1.69 in 1997/1998 and 1.25 in 2002/2003. This indicates that per critically patient there were less severely compromised organ systems in the second period compared to the first.

Table 6 examines the patients that came from the Pretoria area only. The pattern of organ system dysfunction/failure was the same but the numbers were too small to have sufficient power to detect significant differences. The Mortality Index in Pretoria for 1997/1998 was 22.5% and 12.5% for 2002/2003 (OR 0.49, 95% CI 0.15–1.57).

**Table 6:** Comparison of the prevalence of organ system dysfunction/failure per severely ill pregnant women with complications due to hypertension for patients only from the Pretoria area.

Organ system	**1997–1998**				**2002–2003**			
	**SAMM **n = 31	**MD **n = 9	**SAMM+MD **N = 40	**% OSD**	**SAMM **N = 56	**MD **N = 8	**SAMM+MD **N = 64	**% OSD**
Hypovolaemic shock	2	0	2	5.0	3	1	4	6.3
Respiratory failure	7	3	10	25.0	16	2	18	28.1
Cardiac failure	12	4	16	40.0	13	3	16	25.0
Renal failure	6	1	7	17.5	5	1	6	9.4
Liver failure	0	0	0	0.0	1	1	2	3.1
Cerebral complications	8	4	12	30.0	4	3	7	10.9
Haematological dysfunction	9	1	10	25.0	19	1	20	31.3
Immune system failure	0	1	0	2.5	2	2	4	6.3

% OSD – Percentage of severely ill women who developed that organ system dysfunction/failure

Note: A patient can have more than one organ system dysfunction/failure

## Discussion

This report of managing critically ill pregnant women with complications of hypertension in pregnancy during two time periods five years apart has shown a change in pattern of organ system dysfunction/failure. Fewer women developed renal failure and cerebral complications during the later time period. However, other common complications of the respiratory, cardiovascular and immune systems did not seem to be affected by the implementation of the strict protocol.

The possible explanations for the change are better identification of patients with complications and better management, and fewer referrals of critically ill patients from outside areas. By increasing the number of cases identified with improved surveillance, one would have expected a sudden increase in the rate of critically ill women being treated followed by a levelling off. However, there has been a steady increase in the rate from 84/100000 births in 1999, 108/100000 births in 2000 and 138/100000 births in 2001 [14].

The reduction in critically ill patients referred from outside the Pretoria area could also account for the change in our findings. The longer time to get into a tertiary unit is likely to increase the severity of the complications. Thus the shift from 61% of critically ill women being referred in 1997/1998 as opposed to only 48% in 2002/2003 could account for the change. Examining only the cases from the Pretoria area (Table 6), the same shift in pattern is seen although the number of cases was too few to detect significant changes.

The final explanation is that the management of the patients has improved. This is supported by the decreased number of dysfunctional organ systems per patient; it fell from 1.69 in 1997/1998 to 1.25 in 2002/2003. This change may be associated with the introduction and adherence to a strict protocol for managing such critically ill women. It is postulated that using the strict protocol may have prevented further organ systems from becoming involved. The strict protocol was based on the audit in 1997/1998 and the introduction of treatment research findings such as the use of magnesium sulphate for women with severe pre-eclampsia [8]. The feedback system of regular audit meetings to discuss cases, one by one meetings and regular teaching sessions to discuss specific issues confirms the success found in previous randomised trials [6]. The rapid turnover of staff necessitates regular reinforcement of protocols.

One of the limitations of our study was that we used a 'before and after' design to evaluate the effect of the implementation of a strict protocol with no control patients in any of the time periods. Further, the study could not explain the fact that there was no reduction in cardiac and respiratory problems.

The lack of change in the respiratory and cardiovascular system organ dysfunction/failure suggests that there is still significant room for improving the protocol. The confidential enquiries into maternal deaths in the UK revealed that 37% of maternal deaths due to eclampsia or pre-eclampsia during the 1985–1999 period are being ascribed to "pulmonary" [15]. Adult respiratory distress syndrome has been described as contributing factor in 28% [16] of deaths associated with HELLP syndrome. Cardiac and respiratory functions in severe pre-eclampsia are not well understood and future research must attempt to identify the changes that can improve the outcome. The increasing incidence of HIV infection in the area accounts for the rise in immune system failure. This may also affect pulmonary and cardiac function.

The 33% increase of women who were critically ill due to complications of hypertension from the Pretoria area is disturbing. The reason for this is unknown. There has been a steady influx of people into the Gauteng area during the last 5 years. This is confirmed by the 2001 Census [17] and the increase of more than twenty percent of births in the Pretoria area. This influx of many impoverished people looking for employment would be expected to bring more unhealthy people to the region. This will lead to increased pressure on the primary health care services to identify these patients early (at antenatal clinics) and refer appropriately, and increase the pressure on secondary and tertiary services that must deal with the increased load of critically ill patients. Appropriate plans for distribution of resources need to be made to deal with this challenge.

## Conclusion

The strict protocol approach based on the systemic evaluation of critically ill pregnant women due to complications of hypertension has been associated with a reduction in the preventable complications, such as renal failure and cerebral compromise by improved fluid management and blood pressure control. However, there has been no change in the prevalence of cardiac or respiratory failure. Cardiac and respiratory function in women with severe hypertension in pregnancy needs further investigation and strategies need to be developed to improve its management.

## List of Abbreviations

SAMM: Severe acute maternal morbidity

MaMMAS: Maternal Morbidity and Mortality Audit System programme

MD: Maternal Deaths

AIDS: Acquired Immune Deficiency Syndrome

HELLP: Haemolysis, Elevated Liver Enzymes, Low Platelets

AST: Aspartate-amino transferase

UK: United Kingdom

OR: Odds Ratio

MMR: Maternal Mortality Ratio

CTG: Cardio tochography

## Competing interests

The author(s) declare that they have no competing interests.

## Authors' contributions

RCP and PM wrote the initial protocol and coordinated the collection of the SAMM data. FB entered the data on the MaMMAS programme. HL and FB collected the results from the MaMMAS programme. HL, FB and RCP did the statistical analysis. HL wrote the article. RCP and PM substantially improved the manuscript.
